# Presumed Sturge Weber Syndrome in a Haitian Boy: A Case of Delayed Diagnosis

**DOI:** 10.1155/2012/509693

**Published:** 2012-03-27

**Authors:** Gian Paolo Giuliari, Ama Sadaka, Maria Angelica Cortez, Adalgisa Corona

**Affiliations:** ^1^Centro de Cirugía Oftalmológica (CECOF), Caracas, Venezuela; ^2^Ophthalmology Department, Domingo Luciani Hospital, Avenue Francisco de Miranda, Torre Cavendes, Piso 6, Ofic. 6-04, Altamira, Caracas 1070, Venezuela; ^3^“Dr. Elías Santana” Hospital, Santo Domingo, Dominican Republic; ^4^Massachusetts Eye and Ear Infirmary, Boston, MA, USA

## Abstract

*Purpose*. To report an untypical presentation of a presumed Sturge-Weber Syndrome (SWS), and to highlight the indispensable value of thorough clinical examination as primary means for proper diagnosis and management. *Methods*. Chart review. *Results*. A 7 year-old boy, with a long history of ocular symptoms and an unspecified ocular surgery, presents with a painful blind left eye. Based on clinical examination, the suspicion of SWS was raised. The presentation was not typical in the sense that no evident port-wine stain was observed on the face. However, facial asymmetry and gum discoloration were guiding clinical clues to pursue further investigations. Unfortunately, due to poor treatment response, the patient underwent enucleation. Tissue pathology revealed diffuse choroidal hemangiomas, consistent with the diagnosis of SWS. *Conclusion*. SWS presents with hamartomatous malformations and venous dilation affecting the skin, central nervous system and eye. The ocular involvement may vary, with the most common complications being glaucoma, buphthalmos and diffuse choroidal hemangiomas. This case report helps remind physicians of the importance of a thorough clinical examination, and highlights the ophthalmologists' responsibility of examining beyond the eye.

## 1. Introduction

Sturge-Weber syndrome (SWS) is a sporadic, uncommon, congenital neurocutaneous condition consisting of hamartomatous malformations affecting the central nervous system (CNS), skin, and eye usually in a unilateral distribution. It is mainly characterized by facial capillary malformations known as nevus flammus or port-wine stain, ipsilateral leptomeningeal angiomatosis, and possible brain vascular malformations [1, 2]. This condition affects 1 in 20–50,000 live births [3]. The pathophysiology behind it lies in the vascular abnormalities that lead to impaired blood flow [1, 2]. Due to the unilateral nature of the vascular malformation, a somatic mutation or a double-hit model has been proposed as the etiology behind this syndrome [4].

Such conditions stress further the value of a meticulous physical examination that remains crucial for proper patient care despite the advancements in imaging and diagnostic tests. We present the case of a child with untypical presentation where diagnosis and proper management were delayed due to an initial inadequate physical examination and inadequate initial follow-up care.

## 2. Case History

**Figure 1:**
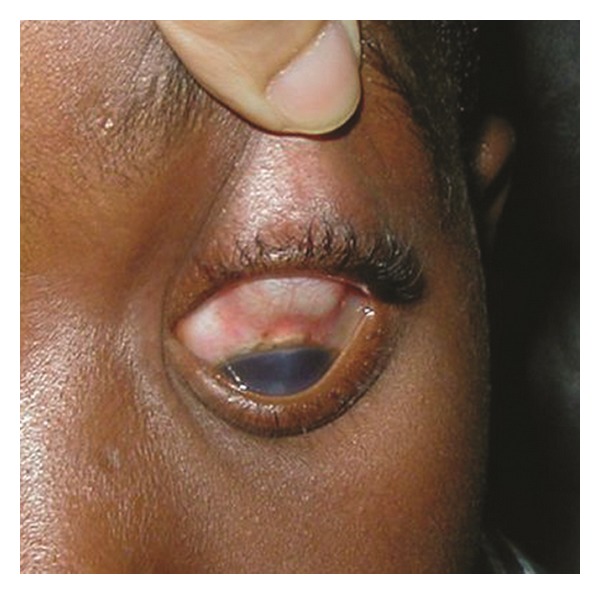
External photograph showing a superior conjunctival bleb in the left eye.

**Figure 2:**
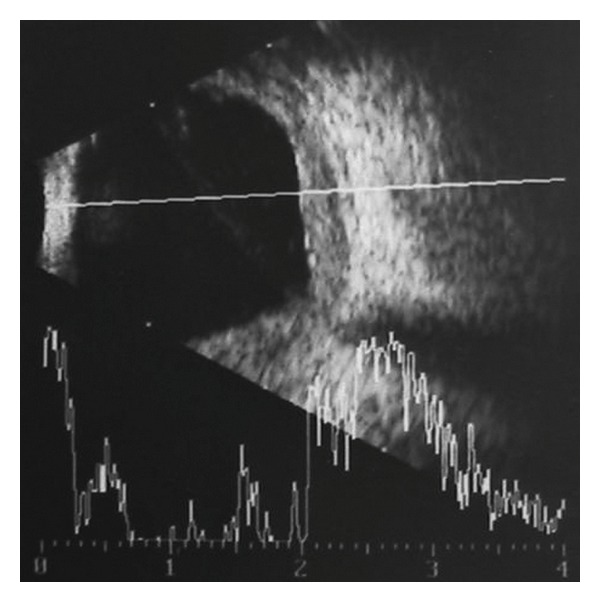
Ultrasound image of the posterior pole of the left eye. Note the thickening of the choroid, compatible with a diffuse choroidal hemangioma.

**Figure 3:**
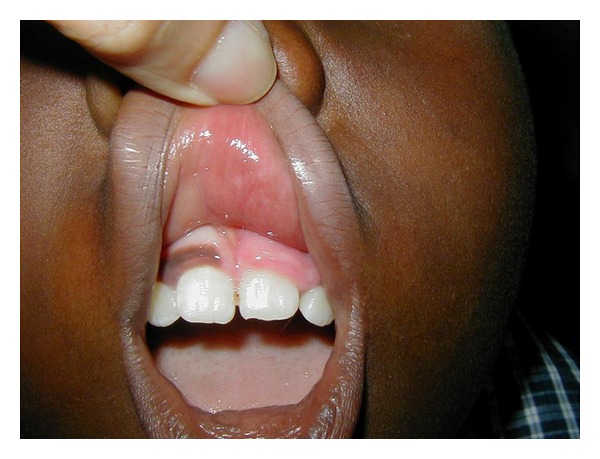
Photograph demonstrating a discoloration of the gum in the left side, consistent with capillary hemangioma.

A 7-year-old boy from Haiti was brought to our clinic at “Dr. Elías Santana” Hospital in Santo Domingo, Dominican Republic, by a Christian missionary. The boy had been complaining for the past 2 years of ocular pain and slowly progressive proptosis in his left eye with no definitive diagnosis. His ocular history was positive for a nonspecified ocular surgery in his left eye 5 years prior to presentation. Examination revealed mild facial asymmetry with evident proptosis of the left eye. Positive eye retropulsion was present. Hertel's exophthalmometry measurements were 15 mm in the right eye and 20 mm in the left eye. Also noted was buphthalmos of his left eye and a large central corneal opacity. His visual acuities were measured as 20/20 in the right eye and no light perception in the left eye. Intraocular pressure was not measured due to poor patient compliance; however, the left eye appeared hard to palpation. Slit-lamp biomicroscopy was normal in the right eye. The corneal largest diameter measured 11 mm in the right eye and 13.5 mm in the left eye. Despite the central corneal opacity in the left eye, that impaired clear visualization of the anterior segment structures, the anterior chamber appeared narrow. Also noted was a superior conjunctival bleb, consistent with a filtrating glaucoma surgery that could have been the procedure performed 5 years previously (Figure 1). Indirect ophthalmoscopy of the right eye was normal. The left eye ocular fundus could not be evaluated due to the media opacity. Hence, an ultrasound was performed to evaluate the state of the posterior segment and revealed diffuse thickening of the choroid, not seen in the right eye (Figure 2). At this point, the diagnosis of SWS was raised; though the typical associated facial nevus flammeus was not present in this patient. Reexamination revealed a discoloration of the gum consistent with capillary hemangioma (Figure 3). A computed tomography (CT) of the brain and orbits was ordered revealing no CNS involvement. Topical medications were given to control the pain and the intraocular pressure in the left eye. Unfortunately, those measures were not sufficient to alleviate the ocular symptoms, necessitating enucleation. Pathology studies of the ocular tissue revealed large congested blood vessels separated by thin intervascular septae, characteristic of a diffuse choroidal hemangioma.

## 3. Discussion

SWS is a syndrome diagnosed on clinical grounds [1–3]. In our patient, at least at first sight, the typical manifestations were not evident. The only apparent clinical sign was a mild facial asymmetry although closer clinical investigation and reassessment revealed the capillary hemangioma in the gums, heightening our suspicion of SWS.

The capillary malformation commonly seen in these patients are frequently present at birth, with an increased chance of brain involvement arising in cases where superior eyelid and forehead lesions are observed [2, 5]. The vascular lesions are usually pink and flat with a tendency to darken and thicken with age [3]. Once diagnosed with SWS, children are usually referred to an ophthalmologist to evaluate the ocular state. Usually, patients without cutaneous manifestations tend not to have ocular disease. Eye involvement may vary with the most common complications being glaucoma, buphthalmos and diffuse choroidal hemangiomas [1, 6]. The ocular clinical signs are usually enlarged, tortuous venous vessels that may affect the conjunctiva, episclera, retina, and choroids. Glaucoma is the most common ophthalmic complication, affecting more than 50% of the patients [6]. If the glaucoma develops during the first year of life, as in our patient's case, buphthalmos develops due to the plasticity of the organs at this stage. The glaucoma may be secondary to the combination of episcleral venous pressure along with anterior chamber anomalies that might impair the normal drainage system of the eye [7]. Ocular hemangiomas of the choroids occur in one-third of the patients, making indirect ophthalmoscopy helpful in making the diagnosis [1, 3]. However, that was not possible with our patient due to the corneal opacity that interfered with the visualization of the posterior segment of the eye. Choroidal hemangiomas is usually flat, frequently involving over half of the fundus from the posterior pole to the equatorial zone. The choroidal involvement tends to grow and thicken with age; however, because of its color resemblance to the normal fundus color during childhood, the hemangioma might be missed. In these cases ultrasound is helpful in revealing the choroidal thickening in one eye and comparing it to the fellow eye.

Management of the intraocular pressure in these patients is usually challenging. In our patient, treatment with topical hypotensive eye drops was prescribed to try to control the intraocular pressure and discomfort in his blind left eye, although this kind of therapy proves to be insufficient in managing the intraocular pressure, requiring surgery most of the times [6].

After relying on the physical exam to diagnose SWS, neuroimaging is usually performed to detect any CNS involvement. In this regard, MRI has been reported to be superior to CT scan in detecting CNS malformation [8]. Given our suspicion of SWS and the limited resources for further investigations, we were able to order a brain CT scan only, that was negative for any brain involvement at that time.

Management of this particular case was challenging due to the untypical presentation, lack of resources for further investigations and followup, inconsistent and unreliable history and lack of any previous medical records. There are many places in the world where health care is not available, and resources to treat and manage patients are limited. There is no doubt that diagnostic tests are invaluable in the diagnosis and management of many diseases, helping not only in monitoring progression, but even in determining disease mechanisms. Nonetheless, the art of clinical examination remains indispensable and central to the management of any patient condition. We believe that this case shows how the clinical suspicion based on the history and physical exam can lead to a proper diagnosis and better patient care.
